# Supplementary material for: Hypocrates is a genetically encoded fluorescent biosensor for (pseudo)hypohalous acids and their derivatives
Source: Nat Commun. 2022 Jan 10;13:171. doi: 10.1038/s41467-021-27796-2 (PMC8748444; doi:10.1038/s41467-021-27796-2)
Supplement: Supplementary file 1 — Supplementary Information [file 41467_2021_27796_MOESM1_ESM.pdf]

Hypocrates is a genetically encoded fluorescent biosensor for (pseudo)hypohalous acids and their derivatives

Supplementary Information

Supplementary figures

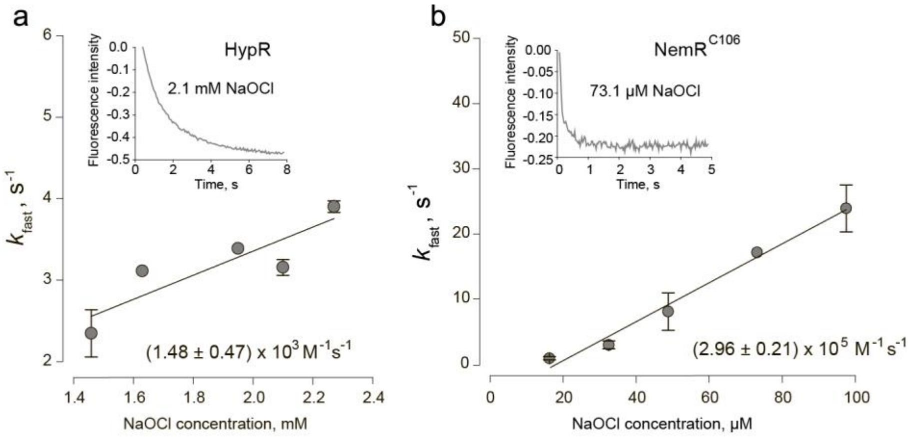

Supplementary Figure 1. $NemR^{C106}$ senses NaOCl 200-fold faster than HypR. The intrinsic Tyr (**a**) and Trp (**b**) fluorescence changes of HypR and $NemR^{C106}$, respectively, at increasing NaOCl concentration are shown as functions of time (inserts). Note, as HypR has no Trp in its sequence, we followed the Tyr fluorescence change. The curves were fitted to a double exponential to obtain the observed rate constants ($k_{obs/fast}$), which were plotted as functions of increasing NaOCl concentration. From the slope of the straight line [$k_{fast} = k_{on}$•[oxidant] + k_{off}], the second-order rate constants were obtained. The second-order rate constant of $NemR^{C106}$ is (2.96 ± 0.21) x 10^5 $M^{-1}s^{-1}$ and the one of HypR is (1.48 ± 0.47) x 10^3 $M^{-1}s^{-1}$. The data are presented as a mean ± SD (for n > 2), n ≥ 1. Source data are provided as a Source Data file.

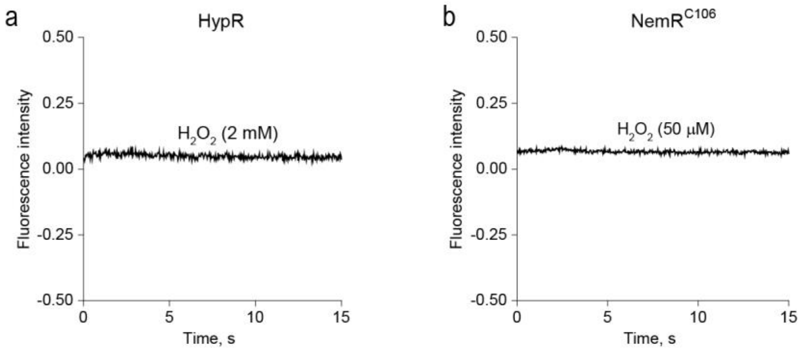

Supplementary Figure 2. HypR and NemR^{C106} do not change their intrinsic fluorescence under H_2O_2 treatment. The changes of intrinsic fluorescence as functions of time for HypR (**a**) and NemR^{C106} (**b**) in the presence of H_2O_2 are shown. To confirm a higher specificity for NaOCl than for H_2O_2, the same concentration as for NaOCl was tested, 50 µM for NemR^{C106} and 2 mM for HypR. Source data are provided as a Source Data file.

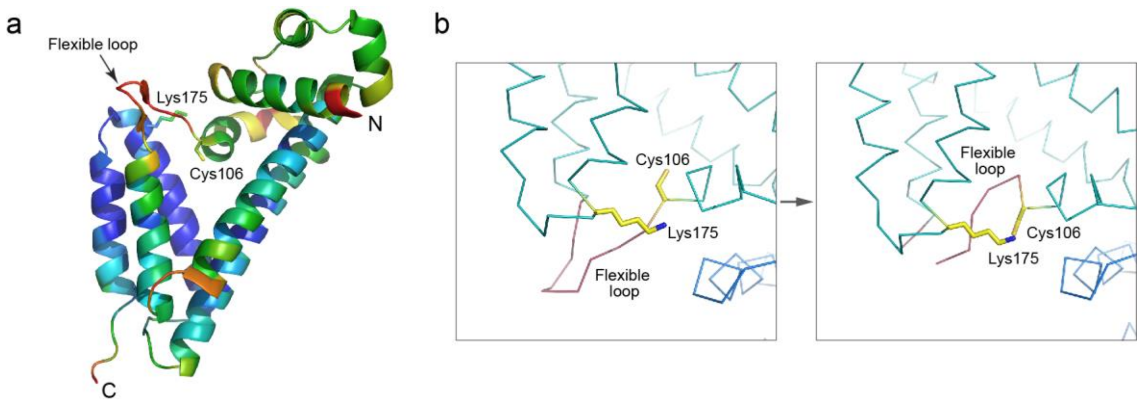

Supplementary Figure 3. The flexible regions of NemR^{C106}. (**a**) The structure of NemR^{C106} (PDB ID: 4YZE [http://doi.org/10.2210/pdb4YZE/pdb]) colored by b-factors (Cα) is shown. The color spectrum shows the flexibility going from the most flexible (red) to the most rigid (blue) regions. (**b**) Different conformations of the Cys106 side chain can be found in the crystallographic asymmetrical unit. It has been suggested that ClO^{-} induced oxidation leads to the transition from the left structure to the right one, affecting the flexible loop organization.

a

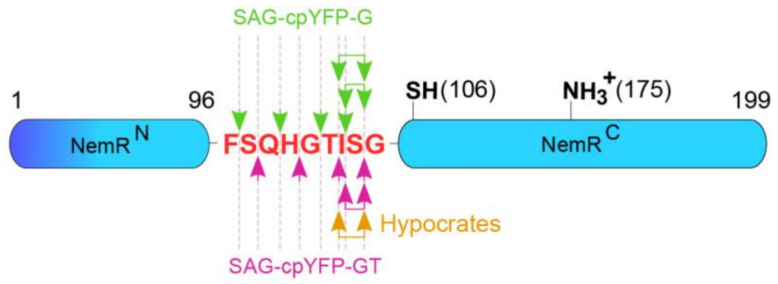

b

Integration position	Max. response amplitude	Brightness at 425 nm (% of Hypocrates)	Brightness at 500 nm (% of Hypocrates)
104-105 (SAG/GT)	~ 1.2	~ 132	~ 82
103-105 (SAG/G)	~ 1.2	~ 71	~ 52
103-105 (SAG/GT)	~ 1.3	~ 143	~ 146
102-105 (SAG/G)	~ 1.3	~ 78	~ 49
102-105 (SAG/GT)	~ 1.6	100	100

c

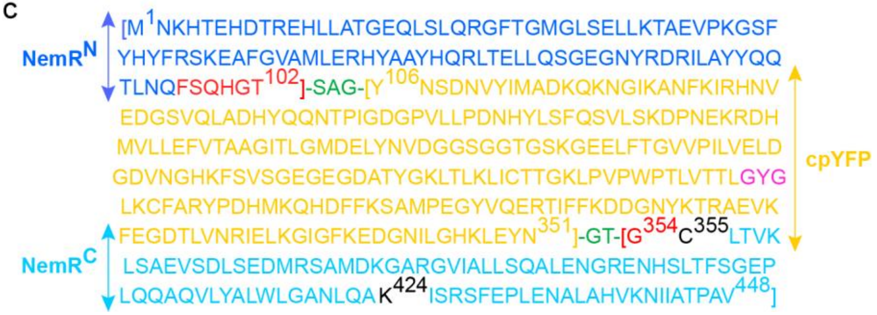

Supplementary Figure 4. (**a**) Different cpYFP insertion positions that were used for the development of the primary versions of NemR-cpYFP biosensor. The numbering on the scheme represents the amino acids corresponding to wild-type NemR. Single and double arrows represent cpYFP insertions without and with deletions, respectively. The green arrows correspond to the versions with SAG/G linkers, the pink arrows correspond to the versions with SAG/GT linkers. The orange arrows correspond to the Hypocrates version. The flexible loop is shown in red. (**b**) Characteristics of the selected primary versions of NemR-cpYFP biosensor (purified proteins) in PBS. (**c**) Hypocrates primary structure. The color legend is the following: blue/cyan – NemR^{C106} derived parts, yellow – cpYFP, red – the flexible loop, green – the linkers connecting NemR^{C106} and cpYFP, black – the key Cys (№106 in NemR^{C106} and №355 in Hypocrates) and Lys (№175 in NemR^{C106} and №424 in Hypocrates) residues, pink – the chromophore triad.

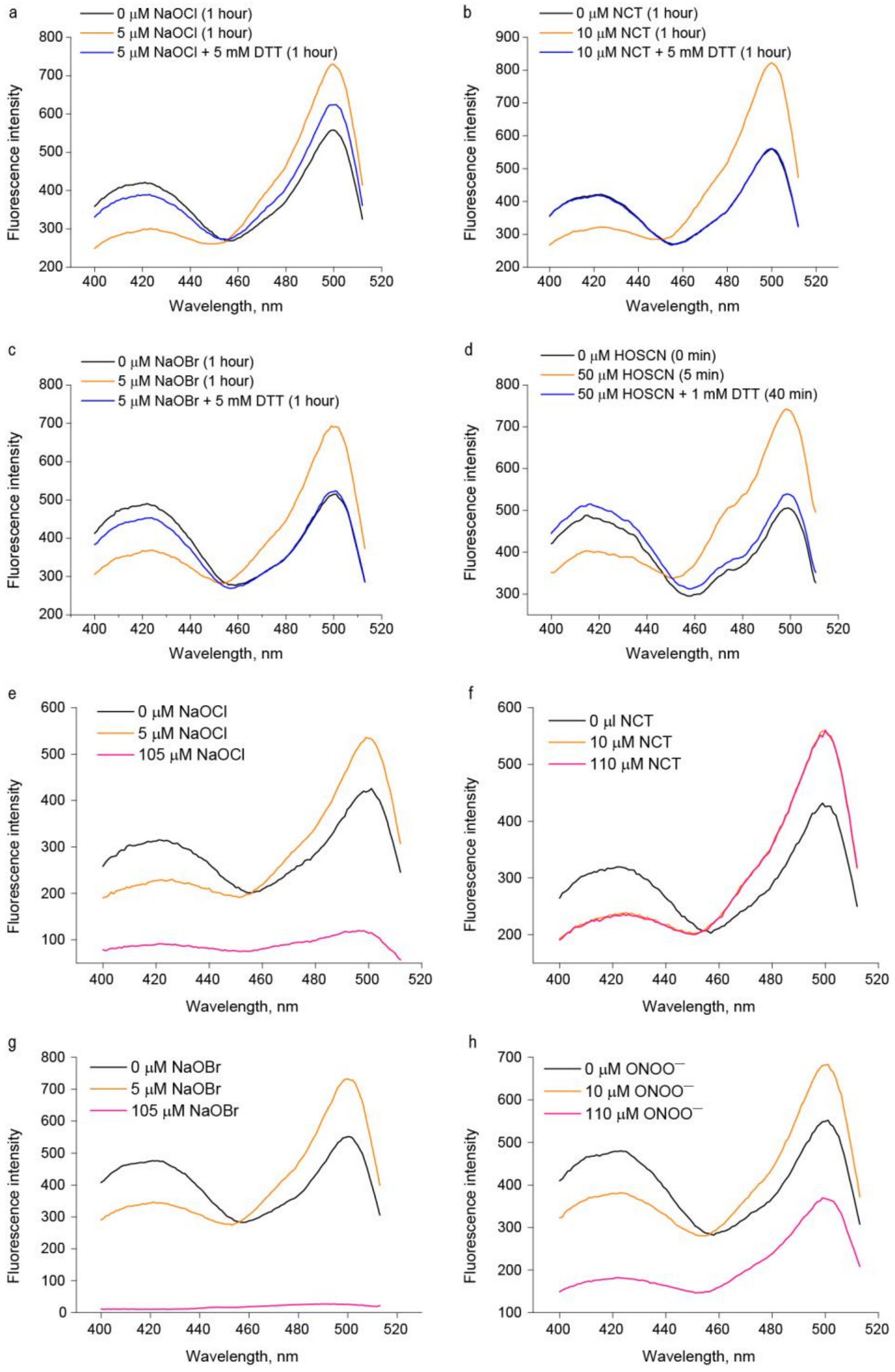

Supplementary Figure 5. (**a-d**) The comparison of Hypocrates fluorescence excitation spectra in the presence of several oxidants: (**a**) NaOCl, (**b**) N-chlorotaurine (NCT), (**c**) NaOBr, and (**d**) HOSCN, as well as the reversibility of responses by DTT. (**e-h**) The degradation resistance of Hypocrates in the presence of high concentrations of several oxidants: (**e**) NaOCl, (**f**) NCT, (**g**) NaOBr, and (**h**) $ONOO^{-}$. Only NCT does not induce fluorescence quenching, apparently due to its lower reactivity and higher specificity toward sulfur-containing amino acids. In all panels, except for **d**, protein concentrations were 0.5 μM. In panel **d**, protein concentration was 2 μM. Panels **a, b, e, f** were recorded in PBS. Panels **c, d, g, h** were recorded in 100 mM sodium phosphate buffer, pH 7.4 to avoid possible OCl^{-} generation. Source data are provided as a Source Data file.

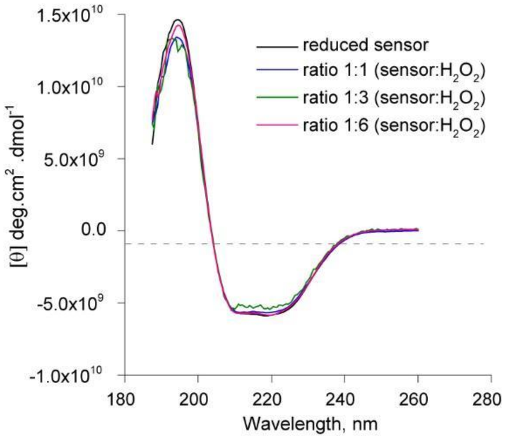

Supplementary Figure 6. Far-UV circular dichroism spectra of reduced and H_2O_2-oxidized sensor: 1:1 ratio, 1:3 ratio and 1:6 ratio Hypocrates vs. oxidant, are shown. Addition of H_2O_2 does not change the overall secondary structure of the biosensor. Source data are provided as a Source Data file.

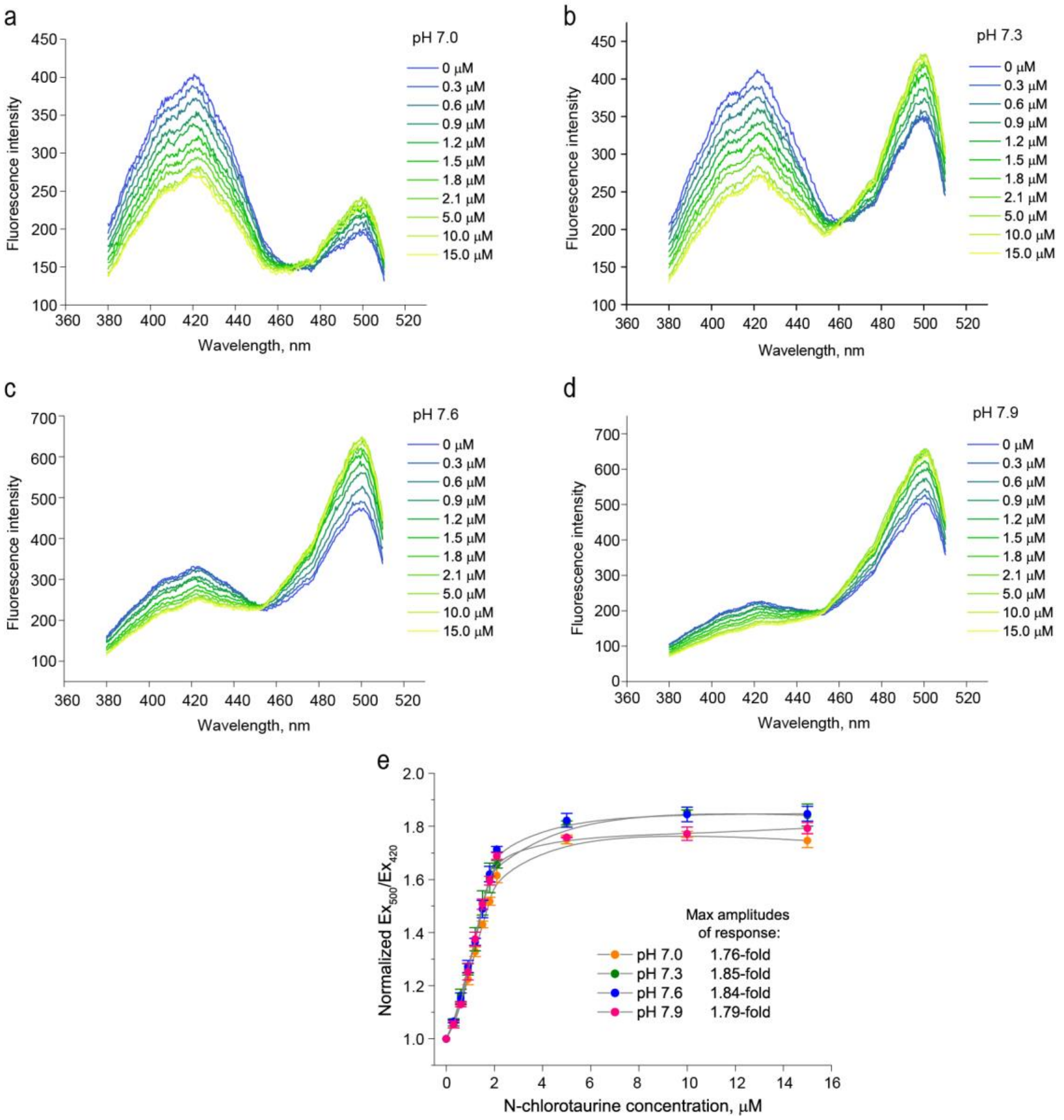

Supplementary Figure 7. (**a-d**) Hypocrates fluorescence excitation spectra in the presence of different N-chlorotaurine concentrations at (**a**) pH 7.0; (**b**) pH 7.3; (**c**) pH 7.6; (**d**) pH 7.9 in 100 mM sodium phosphate buffers. **(e)** The same data represented as titration curves. Protein concentration was 0.5 μM in all cases. The data are presented as a mean ± SEM, n = 3. Source data are provided as a Source Data file.

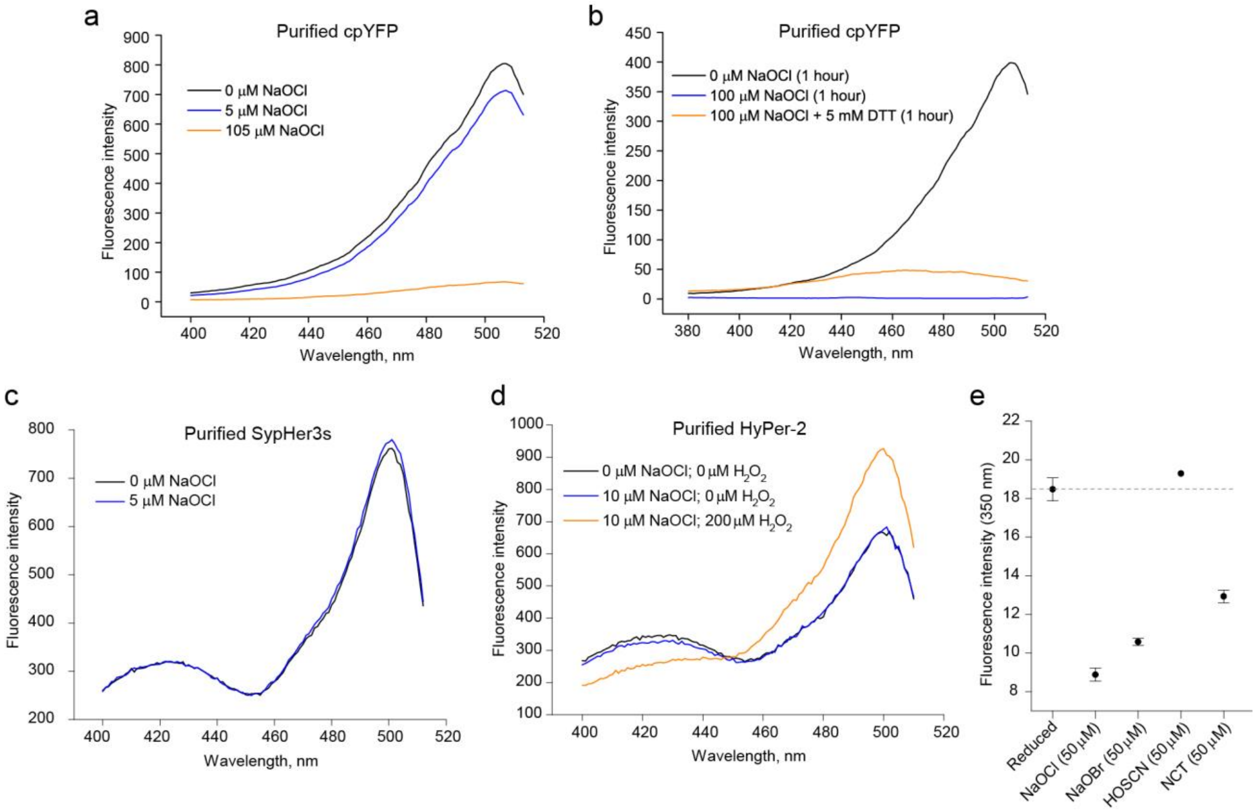

Supplementary Figure 8. The response of different cpYFP-based probes to NaOCl. (**a**) In the presence of low concentrations of NaOCl, the fluorescence excitation spectrum of purified cpYFP shows minor quenching due to apparent protein damaging. With an increase in the concentration of the oxidant, the signal becomes dramatically quenched. (**b**) The spectral changes of cpYFP treated with NaOCl are almost irreversible and cannot be reduced by DTT. (**c**) The fluorescence excitation spectrum of the purified pH biosensor SypHer3s is resistant to NaOCl treatment. (**d**) Purified H_2O_2 biosensor HyPer2 does not react with NaOCl, while the addition of H_2O_2 induces a pronounced ratiometric response. (**e**) The intrinsic Trp fluorescence changes of initial $NemR^{C106}$ in the presence of NaOCl, NaOBr, HOSCN, and N-chlorotaurine (NCT). The data are presented as a mean ± SEM (for n > 2), n = 2 for HOSCN, n = 3 for other compounds. Protein concentration was 0.5 μM in panels **a-d** and 2 μM in panel **e**. Panels **a-d** were recorded in PBS, while panel **e** was recorded in 100 mM sodium phosphate buffer to avoid possible OCl^- generation. Source data are provided as a Source Data file.

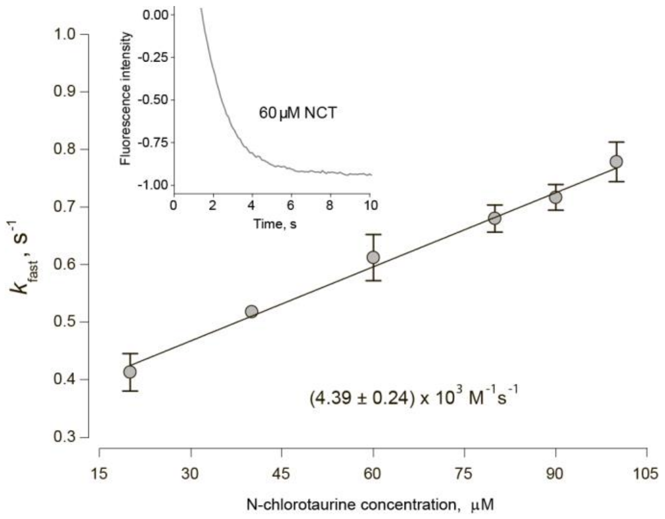

Supplementary Figure 9. The kinetics of $NemR^{C106}$ for N-chlorotaurine (NCT). Changes in intrinsic Trp fluorescence were measured as a function of time (insert). The curves were fitted to a double exponential and k_{fast} values were used as the observed rate constants (k_{obs}), which were plotted as a function of increasing NCT concentration (20-100 µM). From the slope the second-order rate constant of (4.39 ± 0.24) x 10^3 $M^{-1}s^{-1}$ was determined. The data are presented as a mean ± SD (for n > 2), n ≥ 2. Source data are provided as a Source Data file.

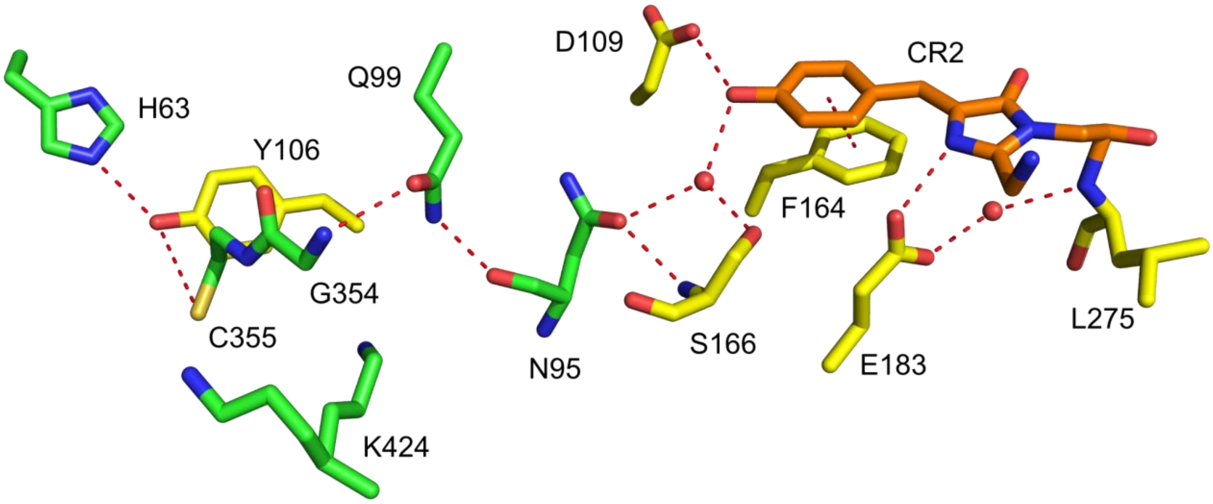

Supplementary Figure 10. Interaction pathway that couples Cys355 and Tyr106 to the hydrogen-bonding network surrounding the chromophore (CR2). The structure of HypocratesCS, in which Ser355 was mutated *in silico* to Cys355, is shown. The residues of cpYFP are shown in yellow; the residues of the NemR^{C106}-sensory domain are shown in green. The most important interactions are shown in red dotted lines. PDB ID: 6ZUI [http://doi.org/10.2210/pdb6ZUI/pdb].

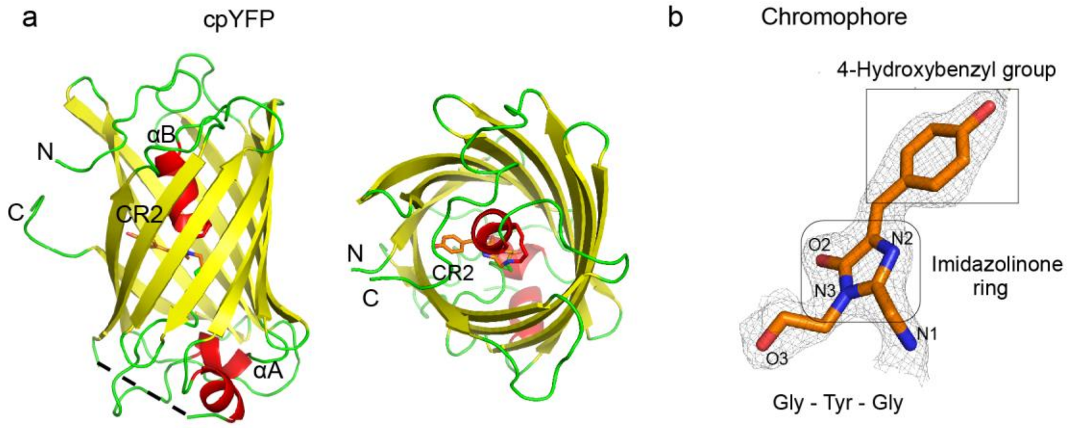

Supplementary Figure 11. The overall cpYFP architecture and the chromophore structure. The β-barrel of cpYFP (side and top views are shown) (**a**) is composed of 11 anti-parallel β-strands (yellow), which are connected via loops (green), and three α-helices (αA, αB and αC - red). The chromophore (CR2) is located next to the C-terminal end of the αB-helix. Due to the high flexibility of an exposed loop (residues 191 to 207 - black dotted line), no defined electron density was observed for this region. (**b**) The chromophore (CR2) structure is shown in orange and is modeled in cis configuration in the electron density map. The 4-hydroxybenzyl group and the imidazolinone ring are indicated by black boxes. PDB ID: 6ZUI [http://doi.org/10.2210/pdb6ZUI/pdb].

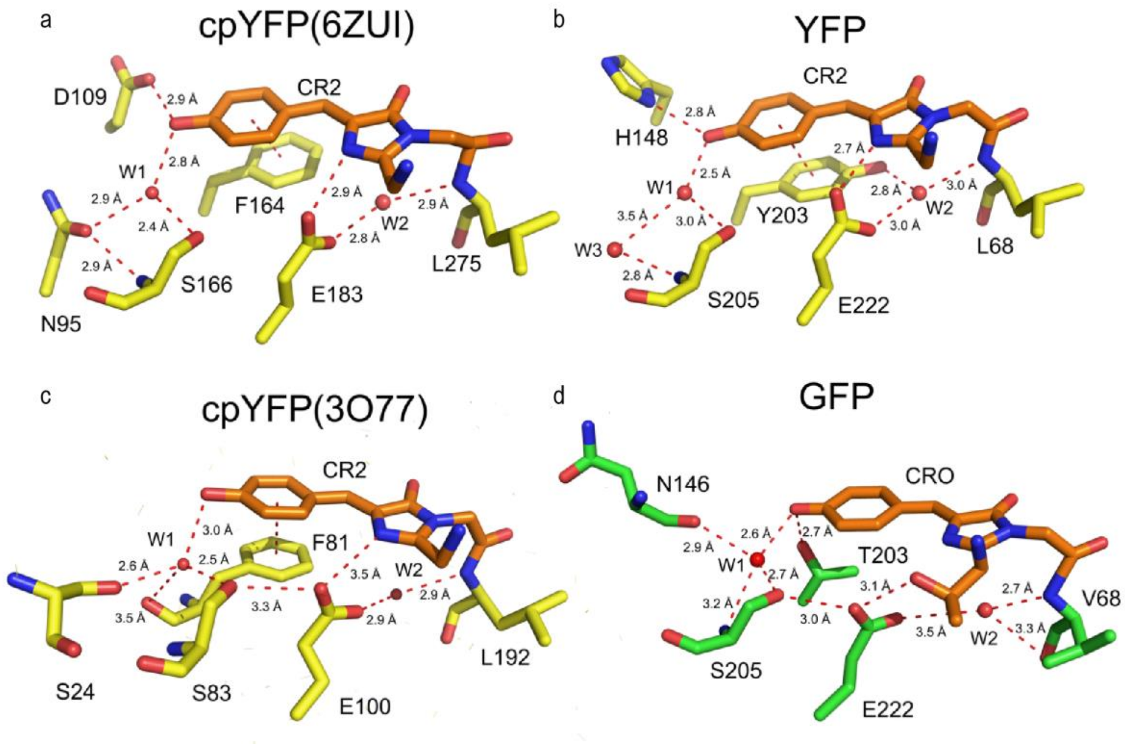

Supplementary Figure 12. The ESPT pathway is different for both cpYFPs, YFP, and GFP. (**a, b**) The CR2 environments of cpYFP (PDB ID: 6ZUI [http://doi.org/10.2210/pdb6ZUI/pdb]) and YFP (PDB ID: 1YFP [http://doi.org/10.2210/pdb1YFP/pdb]) are shown. The positions of the two water molecules (W1 and W2) are conserved. In YFP, the CR2 oxygen is nearly in contact with bulk solvent through the two water molecules (W1 and W3) (panel b). In cpYFP of Hypocrates, the position of W3 is taken by OD1 of N95 (panel a), making the CR2 less solvent-exposed. In YFP, W2 has an extra H-bond with the Y203, which is absent in cpYFP. In cpYFP, the CR2 oxygen will be more deprotonated compared to the YFP because of H-bonding with OD2 of D109. In YFP, partial proton sharing with nitrogen ND1 of H148 will render this CR2 oxygen neutral. (**c**) The structural environment of the CR2 chromophore of the Ca^{2+} sensor Case16 (PDB ID: 3O77 [http://doi.org/10.2210/pdb3O77/pdb]) is shown. S24 links the sensing domain with the chromophore of cpYFP. (**d**) The structural environment of the CRO chromophore of GFP (PDB ID: 2B3P [http://doi.org/10.2210/pdb2B3P/pdb]) is shown. T203 stabilizes a negative charge on the CRO oxygen.

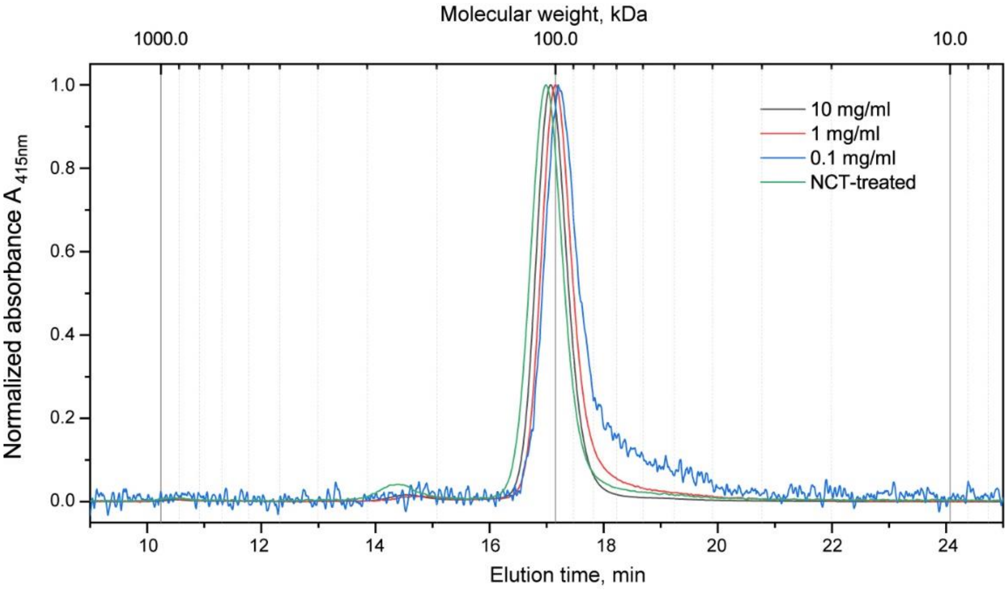

Supplementary Figure 13. Oligomeric state of Hypocrates. Gel filtration elution profiles of reduced Hypocrates at different concentrations and after treatment with N-chlorotaurine (NCT) at 1:20 protein/oxidant ratio. Source data are provided as a Source Data file.

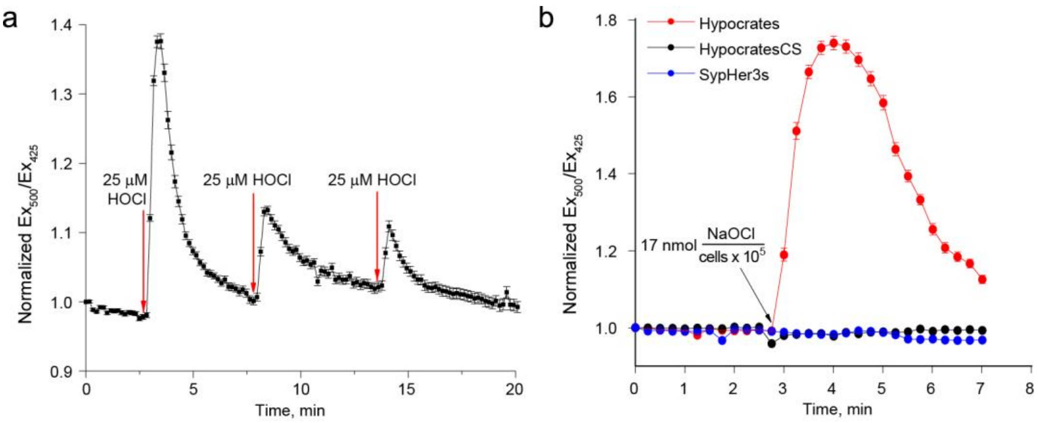

Supplementary Figure 14. Hypocrates in eukaryotic cell culture**. (a)** Hypocrates Ex_{500}/Ex_{425} ratio changes after several serial additions of 10.5 nmol/(10^5 cells) NaOCl (values ± SEM, N = 1 experiment, n = 26 cells). The response of the sensor is reversible; therefore, Hypocrates is capable of registering multiple oxidation/reduction events. **(b)** Hypocrates, HypocratesCS and SypHer3s Ex_{500}/Ex_{425} ratio changes after addition of 17 nmol/(10^5 cells) NaOCl (values ± SEM, N = 3 experiments for Hypocrates and HypocratesCS, N = 2 experiments for SypHer3s, n ≥ 28 cells per experiment). Source data are provided as a Source Data file.

Supplementary Table 1. Optical properties of Hypocrates protein in the fully reduced and the fully oxidized states. Brightness is calculated as a product of extinction coefficient and quantum yield.

Wavelength, nm	Reduced	Oxidized
	Quantum yield	
**425**	~ 0.19	~ 0.15
**500**	~ 0.82	~ 0.83
	Molar extinction coefficient, $M^{-1}cm^{-1}$	
**425**	~ 31200	~ 29600
**500**	~ 12000	~ 16700
	Brightness	
**425**	~ 5900	~ 4400
**500**	~ 9900	~ 13900

Supplementary Table 2. Label free quantification of Trp416 oxidation**.** Relative abundance of the $[M+3H]^{3+}$ precursor ion corresponding to the peptide sequence ENHSLTFSGEPLQQAQVLYAL**W**LGANLQAK was determined from extracted ion chromatograms (XIC) and quantified by the area under the curve (AUC) taking into account the formation of oxindolylalanine (+15.99 Da) or N-formylkynurenine (+31.98 Da) under the different conditions. Oxidation of the Trp residue was confirmed by MS/MS fragmentation of the different parent ions. NCT – N-chlorotaurine.

% Trp oxidation	control	NaOCl	NCT	HOSCN	NaOBr
**mono-oxidation** 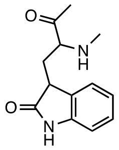	8.1	4.5	5.9	12.1	4.4
**di-oxidation** 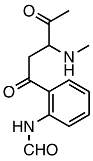	2.0	2.8	1.6	3.9	1.7

Supplementary Table 3. Mass spectrometry summary table. The percentages are calculated based on the number of peptide spectral matches (#PSM) for the chlorination/bromination events and based on Area Under the Curve (AUC) for the Cys and Met oxidations. The methionine oxidation was corrected for mass spectrometric oxidation artefacts using retention times. The raw data are available via ProteomeXchange with identifier PXD029624 [https://doi.org/10.6019/PXD029624]. NCT – N-chlorotaurine.

	**control**	**NaOCl**	**NCT**	**NaOBr**	**HOSCN**
**chlorination**	H232 (16%)	H232 (25%)<br>Y350 (13%)<br>Y246 (7%)<br>Y198 (7%)<br>Y106 (7%)<br>Y82 (2%)	H232 (20%)<br>Y106 (4%)	Y112 (3%)	H232 (28%)
**bromination**				H438 (5%)<br>Y246 (10%)<br>Y299 (4%)<br>Y198 (4%)<br>Y106 (5%)	
**di/tri-oxidation**	C355 (7%)	C355 (76%)	C355 (98%)	C355 (57%)	C355 (5%)
**oxidation**	M59 (3%)<br>M371 (3%)<br>M295 (3%)<br>M179 (0.7%)<br>M29 (8%)<br>M114 (7%)	M59 (44%)<br>M371 (36%)<br>M295 (4%)<br>M179 (35%)<br>M29 (76%)<br>M114 (69%)	M59 (12%)<br>M371 (9%)<br>M295 (2%)<br>M179 (24%)<br>M29 (38%)<br>M114 (76%)	M59 (51%)<br>M371 (24%)<br>M295 (5%)<br>M179 (24%)<br>M29 (67%)<br>M114 (91%)	M59 (7%)<br>M371 (6%)<br>M295 (5%)<br>M179 (5%)<br>M29 (14%)<br>M114 (7%)

The modified His, Tyr, and Met are surface exposed residues. Y106 and C355 are in red and located in close proximity within the sensing domain of Hypocrates.

Supplementary Table 4. X-ray data collection and refinement statistics.

	**HypocratesCS**
**Data collection**	
**Space group**	$C222_1$
**Cell dimensions**	
***a*, *b*, *c* (Å)**	90.23, 95.44, 106.25
**α, β, γ (°)**	90.000, 90.000, 90.000
**Resolution (Å)**	47.72-2.20 (2.27-2.20)*
**R_{merge} (%)**	8.2 (49.5)
**I / σI**	15.8 (2.8
**Spherical completeness (%)**	95.0 (87.9)
**Redundancy**	9.1 (6.2)
**Refinement**	
**Resolution (Å)**	47.72-2.20
**No. reflections**	22261 (2029)
**R_{work} / R_{free}**	19.85/27.30
**No. atoms**	
**Protein**	3299
**Ligand/ion**	na
**Water**	185
***B*-factors**	
**Protein**	42.53
**Ligand/ion**	na
**Water**	43.92
**R.m.s. deviations**	
**Bond lengths (Å)**	0.01
**Bond angles (°)**	1.01

1 crystal was used to solve the HypocratesCS crystal structure
*Values in parentheses are for highest-resolution shell.

Supplementary Table 5. The primers used in this work to engineer NemR-cpYFP versions. “Ins.” means “insertion position”.

Notification	№	Direct primer	№	Reverse primer
**SAG-cpYFP-G**	1	tctgcaggctacaacagcgacaacgtctata tcatggcc	19	accgttgtactccagcttgtgcccca
**SAG-cpYFP-GT**	2	tccgccggctacaacagcgacaacgtctat atcatggcc	20	ggtgccgttgtactccagcttgtgcccca
**NemR edges (pQE)**	3	atatatggatccatgaacaaacacaccgaac atgatactcgc	21	atatataagcttctaaacggcaggcgtcgcaa taatgttttttac
**Ins. 97-98/G**	4	ggcacaagctggagtacaacggtagccaa catggaaccatcagtggttg	22	gttgtcgctgttgtagcctgcagaaaactggtt cagtgtttgctggtaataagcca
**Ins. 98-99/GT**	5	acaagctggagtacaacggcacccaacat ggaaccatcagtggttgcct	23	gctgttgtagccggcggagctaaactggttca gtgtttgctggtaataagc
**Ins. 99-100/G**	6	ggcacaagctggagtacaacggtcatgga accatcagtggttgcctgac	24	gttgtcgctgttgtagcctgcagattggctaaa ctggttcagtgtttgctggtaataag
**Ins. 100-101/GT**	7	acaagctggagtacaacggcaccggaacc atcagtggttgcctgacag	25	gctgttgtagccggcggaatgttggctaaact ggttcagtgtttgctg
**Ins. 101-102/G**	8	ggcacaagctggagtacaacggtaccatca gtggttgcctgacagtaaaactc	26	gttgtcgctgttgtagcctgcagatccatgttg gctaaactggttcagtgtttg
**Ins. 102-103/GT**	9	acaagctggagtacaacggcaccatcagtg gttgcctgacagtaaaactctctg	27	gctgttgtagccggcggaggttccatgttggc taaactggttcagtg
**Ins. 103-104/G**	10	ggcacaagctggagtacaacggtagtggtt gcctgacagtaaaactctctgc	28	gttgtcgctgttgtagcctgcagagatggttcc atgttggctaaactggttcagt
**Ins. 104-105/GT**	11	acaagctggagtacaacggcaccggttgcc tgacagtaaaactctctgcc	29	gctgttgtagccggcggaactgatggttccat gttggctaaactggtt
**Ins. 103-105/G**	12	ggcacaagctggagtacaacggtggttgcc tgacagtaaaactctctgcc	30	gttgtcgctgttgtagcctgcagagatggttcc atgttggctaaactggttcagt
**Ins. 103-105/GT**	13	acaagctggagtacaacggcaccggttgcc tgacagtaaaactctctgcc	31	gctgttgtagccggcggagatggttccatgtt ggctaaactggttcagt
**Ins. 102-105/G**	14	ggcacaagctggagtacaacggtggttgcc tgacagtaaaactctctgcc	32	gttgtcgctgttgtagcctgcagaggttccatg ttggctaaactggttcagtg
**Ins. 102-105/GT**	15	acaagctggagtacaacggcaccggttgcc tgacagtaaaactctctgcc	33	gctgttgtagccggcggaggttccatgttggc taaactggttcagtg
**C355S**	16	ggcaccggtagcctgacagta	34	tactgtcaggctaccggtgcc
**K424A**	17	ctgcaggccgcaatttcgcg	35	cgcgaaattgcggcctgcag
**NemR edges (PCS2)**	18	atatatatcgatgccaccatgaacaaacaca ccgaacatgatactcgc	36	atatattctagactaaacggcaggcgtcgcaa taatgttttttac

CONFIDENTIAL VALIDATION REPORT

WORLDWIDE PDB PROTEIN DATA BANK

Full wwPDB X-ray Structure Validation Report (i)

May 22, 2021 – 03:03 am BST

PDB ID	:	6ZUI
Title	:	Crystal structure of the Cys-Ser mutant of the cpYFP-based biosensor for hypochlorous acid
Deposited on	:	2020-07-23
Resolution	:	2.20 Å(reported)

This is a Full wwPDB X-ray Structure Validation Report.

This report is produced by the wwPDB biocuration pipeline after annotation of the structure.

We welcome your comments at *validation@mail.wwpdb.org*
A user guide is available at
https://www.wwpdb.org/validation/2017/XrayValidationReportHelp
with specific help available everywhere you see the (i) symbol.

The following versions of software and data (see references (i)) were used in the production of this report:

MolProbity	:	4.02b-467
Mogul	:	1.8.5 (274361), CSD as541be (2020)
Xtriage (Phenix)	:	1.13
EDS	:	2.18
Percentile statistics	:	20191225.v01 (using entries in the PDB archive December 25th 2019)
Refmac	:	5.8.0158
CCP4	:	7.0.044 (Gargrove)
Ideal geometry (proteins)	:	Engh & Huber (2001)
Ideal geometry (DNA, RNA)	:	Parkinson et al. (1996)
Validation Pipeline (wwPDB-VP)	:	2.18

1 Overall quality at a glance (i)

The following experimental techniques were used to determine the structure:
X-RAY DIFFRACTION

The reported resolution of this entry is 2.20 Å.

Percentile scores (ranging between 0-100) for global validation metrics of the entry are shown in the following graphic. The table shows the number of entries on which the scores are based.

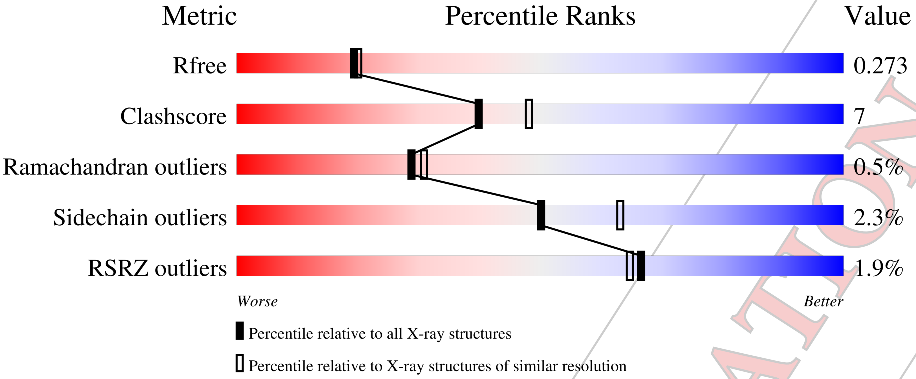

Metric	Whole archive (#Entries)	Similar resolution (#Entries, resolution range(Å))
R_{free}	130704	4898 (2.20-2.20)
Clashscore	141614	5594 (2.20-2.20)
Ramachandran outliers	138981	5503 (2.20-2.20)
Sidechain outliers	138945	5504 (2.20-2.20)
RSRZ outliers	127900	4800 (2.20-2.20)

The table below summarises the geometric issues observed across the polymeric chains and their fit to the electron density. The red, orange, yellow and green segments of the lower bar indicate the fraction of residues that contain outliers for >=3, 2, 1 and 0 types of geometric quality criteria respectively. A grey segment represents the fraction of residues that are not modelled. The numeric value for each fraction is indicated below the corresponding segment, with a dot representing fractions <=5% The upper red bar (where present) indicates the fraction of residues that have poor fit to the electron density. The numeric value is given above the bar.

Mol	Chain	Length	Quality of chain
1	A	446	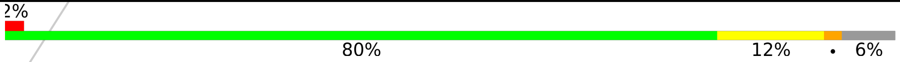

CONFIDENTIAL VALIDATION REPORT

2 Entry composition (i)

There are 2 unique types of molecules in this entry. The entry contains 3503 atoms, of which 0 are hydrogens and 0 are deuteriums.

In the tables below, the ZeroOcc column contains the number of atoms modelled with zero occupancy, the AltConf column contains the number of residues with at least one atom in alternate conformation and the Trace column contains the number of residues modelled with at most 2 atoms.

- Molecule 1 is a protein called HTH-type transcriptional repressor NemR,Green fluorescent protein,Green fluorescent protein,HTH-type transcriptional repressor NemR.

Mol	Chain	Residues	Atoms					ZeroOcc	AltConf	Trace
			Total	C	N	O	S			
1	A	421	3318	2100	573	635	10	1	1	0

There are 36 discrepancies between the modelled and reference sequences:

Chain	Residue	Modelled	Actual	Comment	Reference
A	21	SER	CYS	engineered mutation	UNP P67430
A	98	SER	CYS	engineered mutation	UNP P67430
A	103	SER	-	linker	UNP P67430
A	104	ALA	-	linker	UNP P67430
A	105	GLY	-	linker	UNP P67430
A	109	ASP	HIS	engineered mutation	UNP P42212
A	124	ALA	VAL	engineered mutation	UNP P42212
A	132	VAL	ILE	engineered mutation	UNP P42212
A	164	PHE	THR	engineered mutation	UNP P42212
A	167	VAL	ALA	engineered mutation	UNP P42212
A	192	LEU	HIS	engineered mutation	UNP P42212
A	199	ASN	-	linker	UNP P42212
A	200	VAL	-	linker	UNP P42212
A	201	ASP	-	linker	UNP P42212
A	202	GLY	-	linker	UNP P42212
A	203	GLY	-	linker	UNP P42212
A	204	SER	-	linker	UNP P42212
A	205	GLY	-	linker	UNP P42212
A	206	GLY	-	linker	UNP P42212
A	207	THR	-	linker	UNP P42212
A	208	GLY	-	linker	UNP P42212
A	253	LEU	PHE	engineered mutation	UNP P42212
A	271	LEU	PHE	engineered mutation	UNP P42212
A	273	CR2	SER	chromophore	UNP P42212
A	?	-	TYR	chromophore	UNP P42212
A	?	-	GLY	chromophore	UNP P42212

Continued on next page...

CONFIDENTIAL VALIDATION REPORT

Continued from previous page...

Chain	Residue	Modelled	Actual	Comment	Reference
A	275	LEU	VAL	engineered mutation	UNP P42212
A	276	LYS	GLN	engineered mutation	UNP P42212
A	279	ALA	SER	engineered mutation	UNP P42212
A	336	GLY	ASP	engineered mutation	UNP P42212
A	352	GLY	-	linker	UNP P42212
A	353	THR	-	linker	UNP P42212
A	355	SER	CYS	engineered mutation	UNP P67430
A	365	SER	CYS	engineered mutation	UNP P67430
A	398	SER	CYS	engineered mutation	UNP P67430
A	402	SER	CYS	engineered mutation	UNP P67430

- Molecule 2 is water.

Mol	Chain	Residues	Atoms		ZeroOcc	AltConf
			Total	O		
2	A	185	185	185	0	0

CONFIDENTIAL VALIDATION REPORT

3 Residue-property plots

These plots are drawn for all protein, RNA, DNA and oligosaccharide chains in the entry. The first graphic for a chain summarises the proportions of the various outlier classes displayed in the second graphic. The second graphic shows the sequence view annotated by issues in geometry and electron density. Residues are color-coded according to the number of geometric quality criteria for which they contain at least one outlier: green = 0, yellow = 1, orange = 2 and red = 3 or more. A red dot above a residue indicates a poor fit to the electron density (RSRZ > 2). Stretches of 2 or more consecutive residues without any outlier are shown as a green connector. Residues present in the sample, but not in the model, are shown in grey.

• Molecule 1: HTH-type transcriptional repressor NemR,Green fluorescent protein,Green fluorescent protein,HTH-type transcriptional repressor NemR

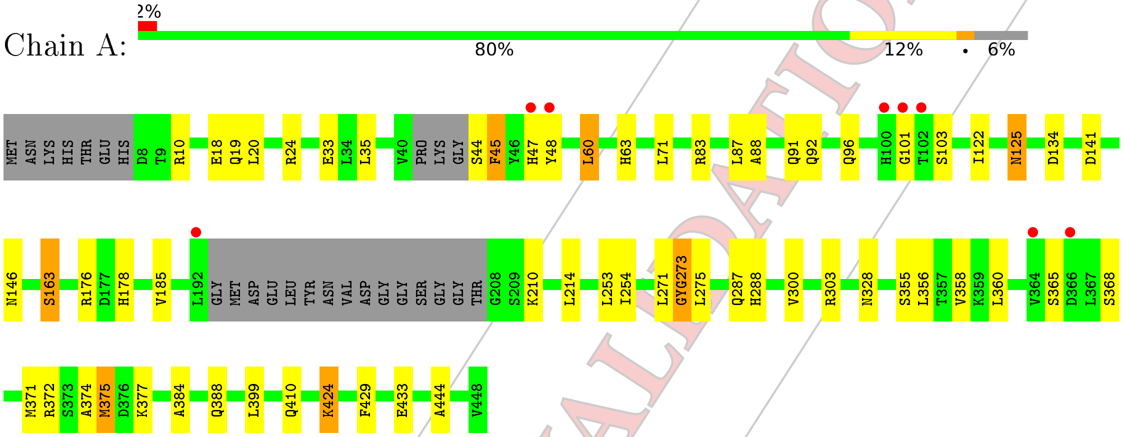

CONFIDENTIAL VALIDATION REPORT

4 Data and refinement statistics (i)

Property	Value	Source
Space group	C 2 2 21	Depositor
Cell constants<br>a, b, c, α, β, γ	90.23Å 95.44Å 106.25Å<br>90.00° 90.00° 90.00°	Depositor
Resolution (Å)	47.72 – 2.20<br>47.72 – 2.20	Depositor<br>EDS
% Data completeness<br>(in resolution range)	94.2 (47.72-2.20)<br>94.2 (47.72-2.20)	Depositor<br>EDS
R_{merge}	0.08	Depositor
R_{sym}	(Not available)	Depositor
$< I/\sigma(I) >$ [1]	2.78 (at 2.20Å)	Xtriage
Refinement program	PHENIX 1.11.1_2575	Depositor
R, R_{free}	0.199 , 0.273<br>0.199 , 0.273	Depositor<br>DCC
R_{free} test set	1136 reflections (5.10%)	wwPDB-VP
Wilson B-factor (Å^2)	34.5	Xtriage
Anisotropy	0.913	Xtriage
Bulk solvent k_{sol}(e/Å^3), B_{sol}(Å^2)	0.30 , 40.2	EDS
L-test for twinning[2]	$< \|L\| >= 0.50$, $< L^2 >= 0.33$	Xtriage
Estimated twinning fraction	No twinning to report.	Xtriage
F_o,F_c correlation	0.95	EDS
Total number of atoms	3503	wwPDB-VP
Average B, all atoms (Å^2)	42.0	wwPDB-VP

Xtriage's analysis on translational NCS is as follows: *The largest off-origin peak in the Patterson function is 5.65% of the height of the origin peak. No significant pseudotranslation is detected.*

[1] Intensities estimated from amplitudes.

[2] Theoretical values of $< |L| >$, $< L^2 >$ for acentric reflections are 0.5, 0.333 respectively for untwinned datasets, and 0.375, 0.2 for perfectly twinned datasets.

CONFIDENTIAL VALIDATION REPORT

5 Model quality (i)

5.1 Standard geometry (i)

Bond lengths and bond angles in the following residue types are not validated in this section: CR2

The Z score for a bond length (or angle) is the number of standard deviations the observed value is removed from the expected value. A bond length (or angle) with $|Z| > 5$ is considered an outlier worth inspection. RMSZ is the root-mean-square of all Z scores of the bond lengths (or angles).

Mol	Chain	Bond lengths		Bond angles	
		RMSZ	#|Z| >5	RMSZ	#|Z| >5
1	A	0.39	0/3366	0.55	0/4541

Chiral center outliers are detected by calculating the chiral volume of a chiral center and verifying if the center is modelled as a planar moiety or with the opposite hand.A planarity outlier is detected by checking planarity of atoms in a peptide group, atoms in a mainchain group or atoms of a sidechain that are expected to be planar.

Mol	Chain	#Chirality outliers	#Planarity outliers
1	A	0	1

There are no bond length outliers.

There are no bond angle outliers.

There are no chirality outliers.

All (1) planarity outliers are listed below:

Mol	Chain	Res	Type	Group
1	A	303	ARG	Peptide

5.2 Too-close contacts (i)

In the following table, the Non-H and H(model) columns list the number of non-hydrogen atoms and hydrogen atoms in the chain respectively. The H(added) column lists the number of hydrogen atoms added and optimized by MolProbity. The Clashes column lists the number of clashes within the asymmetric unit, whereas Symm-Clashes lists symmetry-related clashes.

Mol	Chain	Non-H	H(model)	H(added)	Clashes	Symm-Clashes
1	A	3318	0	3257	47	0
2	A	185	0	0	2	0

Continued on next page...

CONFIDENTIAL VALIDATION REPORT

Continued from previous page...

Mol	Chain	Non-H	H(model)	H(added)	Clashes	Symm-Clashes
All	All	3503	0	3257	47	0

The all-atom clashscore is defined as the number of clashes found per 1000 atoms (including hydrogen atoms). The all-atom clashscore for this structure is 7.

All (47) close contacts within the same asymmetric unit are listed below, sorted by their clash magnitude.

Atom-1	Atom-2	Interatomic distance (Å)	Clash overlap (Å)
1:A:273:CR2:CA3	1:A:275:LEU:N	2.21	1.02
1:A:372:ARG:HH11	1:A:372:ARG:HB3	1.61	0.66
1:A:360:LEU:HB3	1:A:375:MET:HE1	1.78	0.64
1:A:273:CR2:HA31	1:A:275:LEU:N	2.13	0.64
1:A:24:ARG:NH1	1:A:33:GLU:OE2	2.21	0.63
1:A:19:GLN:HE22	1:A:101:GLY:H	1.46	0.62
1:A:122:ILE:HG13	1:A:146:ASN:HB2	1.83	0.60
1:A:273:CR2:C3	1:A:275:LEU:CA	2.77	0.60
1:A:24:ARG:HH12	1:A:134:ASP:HA	1.66	0.59
1:A:273:CR2:O3	1:A:275:LEU:N	2.31	0.59
1:A:19:GLN:NE2	1:A:101:GLY:H	2.02	0.58
1:A:176:ARG:HE	1:A:254:ILE:HG21	1.69	0.58
1:A:371:MET:O	1:A:375:MET:HG2	2.04	0.57
1:A:63:HIS:NE2	1:A:356:LEU:HB3	2.20	0.56
1:A:18:GLU:HG3	1:A:356:LEU:HD22	1.86	0.56
1:A:372:ARG:HB3	1:A:372:ARG:NH1	2.21	0.55
1:A:253:LEU:HD22	1:A:271:LEU:HD22	1.89	0.54
1:A:210:LYS:O	1:A:210:LYS:NZ	2.32	0.52
1:A:96:GLN:HG2	1:A:424[A]:LYS:HE3	1.94	0.50
1:A:275:LEU:HD21	1:A:328:ASN:HB2	1.94	0.50
1:A:88:ALA:O	1:A:92:GLN:HG3	2.11	0.50
1:A:368:SER:O	1:A:372:ARG:HG3	2.12	0.49
1:A:384:ALA:O	1:A:388:GLN:HG2	2.13	0.48
1:A:63:HIS:CD2	1:A:356:LEU:HB3	2.50	0.47
1:A:210:LYS:HZ1	1:A:214:LEU:HG	1.78	0.47
1:A:71:LEU:HD23	1:A:71:LEU:HA	1.80	0.46
1:A:83:ARG:O	1:A:87:LEU:HG	2.16	0.46
1:A:399:LEU:HA	1:A:444:ALA:O	2.17	0.45
1:A:60:LEU:HA	1:A:60:LEU:HD12	1.71	0.45
1:A:146:ASN:HA	1:A:300:VAL:O	2.18	0.44
1:A:125:ASN:ND2	2:A:506:HOH:O	2.50	0.44
1:A:163:SER:O	1:A:185:VAL:HA	2.18	0.44
1:A:88:ALA:HA	1:A:91:GLN:HB3	2.00	0.44

Continued on next page...

CONFIDENTIAL VALIDATION REPORT

Continued from previous page...

Atom-1	Atom-2	Interatomic distance (Å)	Clash overlap (Å)
1:A:377:LYS:HA	1:A:377:LYS:HE2	1.99	0.44
1:A:375:MET:HG2	1:A:375:MET:H	1.53	0.44
1:A:44:SER:HA	1:A:47:HIS:NE2	2.33	0.43
1:A:24:ARG:NH1	1:A:134:ASP:HA	2.33	0.43
1:A:178:HIS:HB2	1:A:254:ILE:HD13	2.01	0.43
1:A:287:GLN:HG2	1:A:288:HIS:CD2	2.54	0.42
1:A:101:GLY:HA2	2:A:601:HOH:O	2.19	0.42
1:A:375:MET:HE2	1:A:375:MET:HB3	1.64	0.42
1:A:35:LEU:HD11	1:A:45:PHE:HB2	2.01	0.41
1:A:60:LEU:HD23	1:A:374:ALA:HB3	2.02	0.41
1:A:355:SER:OG	1:A:358:VAL:HG22	2.20	0.41
1:A:429:PHE:O	1:A:433:GLU:HG3	2.20	0.40
1:A:10:ARG:HG3	1:A:48:TYR:CD1	2.56	0.40
1:A:20:LEU:HD12	1:A:20:LEU:HA	1.88	0.40

There are no symmetry-related clashes.

5.3 Torsion angles ⓘ

5.3.1 Protein backbone ⓘ

In the following table, the Percentiles column shows the percent Ramachandran outliers of the chain as a percentile score with respect to all X-ray entries followed by that with respect to entries of similar resolution.

The Analysed column shows the number of residues for which the backbone conformation was analysed, and the total number of residues.

Mol	Chain	Analysed	Favoured	Allowed	Outliers	Percentiles
1	A	413/446 (93%)	404 (98%)	7 (2%)	2 (0%)	29 31

All (2) Ramachandran outliers are listed below:

Mol	Chain	Res	Type
1	A	365	SER
1	A	45	PHE

CONFIDENTIAL VALIDATION REPORT

5.3.2 Protein sidechains (i)

In the following table, the Percentiles column shows the percent sidechain outliers of the chain as a percentile score with respect to all X-ray entries followed by that with respect to entries of similar resolution.

The Analysed column shows the number of residues for which the sidechain conformation was analysed, and the total number of residues.

Mol	Chain	Analysed	Rotameric	Outliers	Percentiles	
1	A	353/371 (95%)	344 (98%)	9 (2%)	47	60

All (9) residues with a non-rotameric sidechain are listed below:

Mol	Chain	Res	Type
1	A	60	LEU
1	A	103	SER
1	A	125	ASN
1	A	141	ASP
1	A	163	SER
1	A	375	MET
1	A	410	GLN
1	A	424[A]	LYS
1	A	424[B]	LYS

Sometimes sidechains can be flipped to improve hydrogen bonding and reduce clashes. All (1) such sidechains are listed below:

Mol	Chain	Res	Type
1	A	19	GLN

5.3.3 RNA (i)

There are no RNA molecules in this entry.

5.4 Non-standard residues in protein, DNA, RNA chains (i)

1 non-standard protein/DNA/RNA residue is modelled in this entry.

In the following table, the Counts columns list the number of bonds (or angles) for which Mogul statistics could be retrieved, the number of bonds (or angles) that are observed in the model and the number of bonds (or angles) that are defined in the Chemical Component Dictionary. The Link column lists molecule types, if any, to which the group is linked. The Z score for a bond length (or angle) is the number of standard deviations the observed value is removed from the

CONFIDENTIAL VALIDATION REPORT

expected value. A bond length (or angle) with $|Z| > 2$ is considered an outlier worth inspection. RMSZ is the root-mean-square of all Z scores of the bond lengths (or angles).

Mol	Type	Chain	Res	Link	Bond lengths			Bond angles		
					Counts	RMSZ	$\#\|Z\| > 2$	Counts	RMSZ	$\#\|Z\| > 2$
1	CR2	A	273	1	20,20,21	3.95	5 (25%)	25,27,29	4.42	11 (44%)

In the following table, the Chirals column lists the number of chiral outliers, the number of chiral centers analysed, the number of these observed in the model and the number defined in the Chemical Component Dictionary. Similar counts are reported in the Torsion and Rings columns. '-' means no outliers of that kind were identified.

Mol	Type	Chain	Res	Link	Chirals	Torsions	Rings
1	CR2	A	273	1	-	2/6/25/26	0/2/2/2

All (5) bond length outliers are listed below:

Mol	Chain	Res	Type	Atoms	Z	Observed(Å)	Ideal(Å)
1	A	273	CR2	CB2-CA2	15.96	1.48	1.35
1	A	273	CR2	CA2-C2	-4.83	1.43	1.48
1	A	273	CR2	C2-N3	-2.97	1.32	1.39
1	A	273	CR2	O2-C2	2.57	1.28	1.23
1	A	273	CR2	C1-N2	2.54	1.37	1.32

All (11) bond angle outliers are listed below:

Mol	Chain	Res	Type	Atoms	Z	Observed(^{o})	Ideal(^{o})
1	A	273	CR2	O2-C2-CA2	-15.24	122.40	130.96
1	A	273	CR2	CA2-C2-N3	12.57	109.32	103.37
1	A	273	CR2	C2-N3-C1	-4.69	105.70	107.99
1	A	273	CR2	O3-C3-CA3	-3.53	115.73	126.39
1	A	273	CR2	C2-CA2-N2	-3.30	106.62	108.93
1	A	273	CR2	C1-CA1-N1	-3.28	105.60	112.85
1	A	273	CR2	CB2-CA2-C2	2.76	125.57	122.28
1	A	273	CR2	CE1-CD1-CG2	-2.45	118.06	121.25
1	A	273	CR2	CA3-N3-C2	2.37	129.24	123.80
1	A	273	CR2	CD2-CG2-CD1	2.25	120.97	117.64
1	A	273	CR2	CA3-N3-C1	-2.02	125.06	127.86

There are no chirality outliers.

All (2) torsion outliers are listed below:

CONFIDENTIAL VALIDATION REPORT

Mol	Chain	Res	Type	Atoms
1	A	273	CR2	C3-CA3-N3-C2
1	A	273	CR2	C3-CA3-N3-C1

There are no ring outliers.

1 monomer is involved in 4 short contacts:

Mol	Chain	Res	Type	Clashes	Symm-Clashes
1	A	273	CR2	4	0

5.5 Carbohydrates (i)

There are no monosaccharides in this entry.

5.6 Ligand geometry (i)

There are no ligands in this entry.

5.7 Other polymers (i)

There are no such residues in this entry.

5.8 Polymer linkage issues (i)

The following chains have linkage breaks:

Mol	Chain	Number of breaks
1	A	1

All chain breaks are listed below:

Model	Chain	Residue-1	Atom-1	Residue-2	Atom-2	Distance (Å)
1	A	273:CR2	C3	275:LEU	N	1.71

CONFIDENTIAL VALIDATION REPORT

6 Fit of model and data (i)

6.1 Protein, DNA and RNA chains (i)

In the following table, the column labelled '#RSRZ> 2' contains the number (and percentage) of RSRZ outliers, followed by percent RSRZ outliers for the chain as percentile scores relative to all X-ray entries and entries of similar resolution. The OWAB column contains the minimum, median, 95th percentile and maximum values of the occupancy-weighted average B-factor per residue. The column labelled 'Q< 0.9' lists the number of (and percentage) of residues with an average occupancy less than 0.9.

Mol	Chain	Analysed	<RSRZ>	#RSRZ>2			OWAB(Å^2)	Q<0.9
1	A	420/446 (94%)	-0.17	8 (1%)	66	65	26, 41, 57, 72	16 (3%)

All (8) RSRZ outliers are listed below:

Mol	Chain	Res	Type	RSRZ
1	A	48	TYR	3.2
1	A	102	THR	3.1
1	A	192	LEU	2.9
1	A	366	ASP	2.8
1	A	100	HIS	2.4
1	A	47	HIS	2.4
1	A	364	VAL	2.1
1	A	101	GLY	2.1

6.2 Non-standard residues in protein, DNA, RNA chains (i)

In the following table, the Atoms column lists the number of modelled atoms in the group and the number defined in the chemical component dictionary. The B-factors column lists the minimum, median, 95th percentile and maximum values of B factors of atoms in the group. The column labelled 'Q< 0.9' lists the number of atoms with occupancy less than 0.9.

Mol	Type	Chain	Res	Atoms	RSCC	RSR	B-factors(Å^2)	Q<0.9
1	CR2	A	273	19/20	0.97	0.12	23,29,33,35	0

6.3 Carbohydrates (i)

There are no monosaccharides in this entry.

CONFIDENTIAL VALIDATION REPORT

6.4 Ligands (i)

There are no ligands in this entry.

6.5 Other polymers (i)

There are no such residues in this entry.

CONFIDENTIAL VALIDATION REPORT
