# Supplementary material for: Hypocrates is a genetically encoded fluorescent biosensor for (pseudo)hypohalous acids and their derivatives
Source: Nat Commun. 2022 Jan 10;13:171. doi: 10.1038/s41467-021-27796-2 (PMC8748444; doi:10.1038/s41467-021-27796-2)
Supplement: Supplementary file 3 — Reporting Summary [file 41467_2021_27796_MOESM3_ESM.pdf]

nature portfolio

Corresponding author(s): Joris Messens, Dmitry S. Bilan, Vsevolod V. Belousov

Last updated by author(s): Dec 6, 2021

Reporting Summary

Nature Portfolio wishes to improve the reproducibility of the work that we publish. This form provides structure for consistency and transparency in reporting. For further information on Nature Portfolio policies, see our Editorial Policies and the Editorial Policy Checklist.

Statistics

For all statistical analyses, confirm that the following items are present in the figure legend, table legend, main text, or Methods section.

n/a	Confirmed	
☐	☒	The exact sample size (n) for each experimental group/condition, given as a discrete number and unit of measurement
☐	☒	A statement on whether measurements were taken from distinct samples or whether the same sample was measured repeatedly
☐	☒	The statistical test(s) used AND whether they are one- or two-sided<br>*Only common tests should be described solely by name; describe more complex techniques in the Methods section.*
☐	☒	A description of all covariates tested
☐	☒	A description of any assumptions or corrections, such as tests of normality and adjustment for multiple comparisons
☐	☒	A full description of the statistical parameters including central tendency (e.g. means) or other basic estimates (e.g. regression coefficient) AND variation (e.g. standard deviation) or associated estimates of uncertainty (e.g. confidence intervals)
☐	☒	For null hypothesis testing, the test statistic (e.g. F, t, r) with confidence intervals, effect sizes, degrees of freedom and P value noted<br>*Give P values as exact values whenever suitable.*
☒	☐	For Bayesian analysis, information on the choice of priors and Markov chain Monte Carlo settings
☒	☐	For hierarchical and complex designs, identification of the appropriate level for tests and full reporting of outcomes
☒	☐	Estimates of effect sizes (e.g. Cohen's d, Pearson's r), indicating how they were calculated

Our web collection on statistics for biologists contains articles on many of the points above.

Software and code

Policy information about availability of computer code

Data collection

Tecan Infinite 200 PRO was controlled by Tecan i-control 1.11.1.0 software.
Leica DMI 6000 microscope was controlled by LAS X 2.6.4.8702 software.
Varian Cary Eclipse Fluorescence Spectrophotometer was controlled by Cary Eclipse Scan 1.1(132)/Kinetics 1.1(133) Application.
Varian Cary 5000 Spectrophotometer was controlled by Varian UV Scan Application 3.00(339).
AKTA Pure chromatography system was controlled by UNICORN 6.3.0.731 software.
LS55 luminescence spectrophotometer was controlled by FL WinLAB 4.00.03 software.
Applied Photophysics SV20 stopped-flow spectrometer was controlled by Prodata SX 2.5.0 software.
SpectraMax iD5 plate reader was controlled by Softmax Pro 7.1 software.
Jasco J-715 spectropolarimeter was controlled by J-700 1.07.00 software.
The x-ray data collection software at Soleil Proxima 1 is MXCuBE Qt3 v 2.1.
Nikon Instruments Inc. ECLIPSE Ti2 inverted microscope was controlled by NIS-Elements 5.21.03 software.
Agilent 1260 LC system was controlled by Agilent OpenLAB CDS ChemStation Edition C.01.07 SR3 software.
Orbitrap Fusion Lumos mass spectrometer was controlled by XCalibur 4.3 and LUMOS tune application 3.3.
Zeiss Axio Observer Z1 inverted microscope was controlled by Metamorph Premier 7.8 software.

Data analysis	In vitro experiments: OriginPro 9.0 and GraphPad Prism8 for calculations.<br>Crystal structure determination: XDS 0.6.5.5 for data processing, Phaser 2.7.16 in the Phenix gui for molecular replacement, Phenix.refinement 1.11.1_2575 for refinement, Coot 0.8.9.2 EL for manual model building.<br>Mass spectrometry data: Proteome Discoverer 2.4 and Freestyle 1.6 from Thermo Scientific.<br>HeLa Kyoto experiments: Fiji 2.0.0-rc-69/1.52p/Java 1.8.0_172 for image processing, MS Excel 2016 and OriginPro 9.0 for calculations.<br>Experiments with neutrophils: Fiji 2.0.0-rc-69/1.52p/Java 1.8.0_172 for image processing, MS Excel 2016 and OriginPro 9.0 for calculations.<br>Danio rerio experiments: Fiji 2.0.0-rc-69/1.52p/Java 1.8.0_172 for image processing, MS Excel 2016 and GraphPad Prism8 for calculations.

For manuscripts utilizing custom algorithms or software that are central to the research but not yet described in published literature, software must be made available to editors and reviewers. We strongly encourage code deposition in a community repository (e.g. GitHub). See the Nature Portfolio guidelines for submitting code & software for further information.

Data

Policy information about availability of data

All manuscripts must include a data availability statement. This statement should provide the following information, where applicable:

- Accession codes, unique identifiers, or web links for publicly available datasets
- A description of any restrictions on data availability
- For clinical datasets or third party data, please ensure that the statement adheres to our policy

The X-ray crystal structure of HypocratesCS was deposited in the protein data bank under accession code 6ZUI. The entry has been assigned the following PDB DOI: https://doi.org/10.2210/pdb6ZUI/pdb. The mass spectrometry data have been deposited to the ProteomeXchange Consortium via the PRIDE partner repository with the dataset identifier PXD029624 and https://doi.org/10.6019/PXD029624. Source data are provided with this paper. The raw data are available upon reasonable request from the corresponding authors.

Field-specific reporting

Please select the one below that is the best fit for your research. If you are not sure, read the appropriate sections before making your selection.

☒ Life sciences ☐ Behavioural & social sciences ☐ Ecological, evolutionary & environmental sciences

For a reference copy of the document with all sections, see nature.com/documents/nr-reporting-summary-flat.pdf

Life sciences study design

All studies must disclose on these points even when the disclosure is negative.

Sample size	For in vitro experiments, no sample size calculation protocols were used. The minimum number of independent measurements was 3 for most assays since the used equipment provided sufficient level of data consistency as can be seen in the Source Data file. For some tests, in which the relationships among variables were investigated, the points were measured in single replicas; however, in such cases the desired accuracy was achieved due to sufficient number of steps. Some experiments were performed in a single replica (e.g. the registration of CD spectra or fluorescence excitation spectra in bacteria) since pronounced qualitative effects were investigated. These experiments were never used to construct any quantitative conclusions, and their aim was to visually demonstrate the main trends in the behavior of the proteins.<br>For HeLa Kyoto experiments, no sample size calculation protocols were used. The minimum number of independent experiments was 1 (usually, 3) and the minimum number of cells per measurement was 25 or more. We presume that such sample sizes are sufficient, because we observed relatively low variability between individual cells as can be seen in the Source Data file. Generally, the selected sample sizes are common in the field.<br>For experiments with neutrophils, no sample size calculation protocols were used. In each case, the number of independent measurements was 3 and the total number of cells per experiment was 35. As can be seen in the Source Data file, dispersion was relatively weak within a given condition. Generally, the selected sample sizes are common in the field.<br>For Danio rerio experiments, no statistical methods were used to predetermine sample size. We always mounted 3 to 4 larvae per experiment for imaging and we reproduced the experiment 3 or 4 times. Some larvae are out of focus and cannot be used for imaging. As shown in the Source Data file, dispersion was weak within a given condition, and difference was statistically significant between conditions.
Data exclusions	For in vitro experiments, no data were excluded.<br>For HeLa Kyoto experiments, no data were excluded.<br>For experiments with neutrophils, no data were excluded.<br>For Danio rerio experiments, no data were excluded. For imaging, embryos were mounted in agarose. Animals out of focus were discarded.
Replication	In vitro experiments were mostly performed in duplicates or triplicates. All replication attempts were successful and provided matching results. Moreover, many experiments were independently performed in two different laboratories and still provided a good level of data consistency.<br>HeLa Kyoto experiments were mostly performed in triplicates. All replication attempts were successful and provided matching results.<br>Experiments with neutrophils were performed in triplicates. All replication attempts were successful and provided matching results.<br>For Danio rerio experiments, all the findings were replicated. For the Hypocrates sensor, the data correspond to 4 independent experiments performed within 2 months of work. We checked with ANOVA followed by Tukey's post hoc test whether independent experiments gave significantly different results, which was never the case. Consequently, independent experiments were pooled. For the HyPerRed sensor, 3 independent experiments were performed, and treated as above.
Randomization	No randomization was required for in vitro experiments. Since tested aliquots were taken from the concentrated protein solution which was

	obtained according to a fixed protocol, we can assume that the in vitro samples were randomly allocated. In HeLa Kyoto experiments, the imaged cells were plated from the same culture in the same conditions; therefore, no randomization was required. The cell line we use is isogenic and consequently cells were randomly allocated. In each experiment with neutrophils, the imaged cells were taken from the total mixture of cells collected from all the volunteers at the beginning of the day and sensors were tested in parallel with controls; therefore, no randomization was required. Since the cells from all participants were present in each sample, we can assume that these cells were randomly allocated. For Danio rerio experiments, the animals were picked from a pool of animals. The strain we use is isogenic and consequently larvae were randomly allocated.
Blinding	Blinding procedures were not required for in vitro experiments. In these tests, the responses of the proteins were measured by the optical equipment automatically. Since all data were processed according to the same protocol, any blinding procedures were not required. In HeLa Kyoto experiments, the responses of the sensors were measured by the optical equipment automatically. Since all images were processed according to the same protocol, any blinding procedures were not required. In experiments with neutrophils, the responses of the sensors were measured by the optical equipment automatically. Since all images were processed according to the same protocol, any blinding procedures were not required. For Danio rerio experiments, the investigators were not blinded to group allocation during data collection which was conducted according to a standard protocol for all animals; however, all quantifications were performed in blind.

Reporting for specific materials, systems and methods

We require information from authors about some types of materials, experimental systems and methods used in many studies. Here, indicate whether each material, system or method listed is relevant to your study. If you are not sure if a list item applies to your research, read the appropriate section before selecting a response.

Materials & experimental systems

n/a	Involved in the study
☒	☐ Antibodies
☐	☒ Eukaryotic cell lines
☒	☐ Palaeontology and archaeology
☐	☒ Animals and other organisms
☐	☒ Human research participants
☒	☐ Clinical data
☒	☐ Dual use research of concern

Methods

n/a	Involved in the study
☒	☐ ChIP-seq
☒	☐ Flow cytometry
☒	☐ MRI-based neuroimaging

Eukaryotic cell lines

Policy information about cell lines

Cell line source(s)	HeLa-Kyoto EMBL RRID:CVCL_1922.
Authentication	None of the cell lines have been authenticated.
Mycoplasma contamination	HeLa Kyoto were tested negative for mycoplasma contamination.
Commonly misidentified lines (See ICLAC register)	None commonly misidentified lines were used.

Animals and other organisms

Policy information about studies involving animals; ARRIVE guidelines recommended for reporting animal research

Laboratory animals	Danio rerio (male and female), 2-day age, wild-type Tübingen (TU) strain.
Wild animals	No wild animals were used in this study.
Field-collected samples	No field-collected samples were used in this study.
Ethics oversight	French Ministry of Agriculture (n°C75-05-12).

Note that full information on the approval of the study protocol must also be provided in the manuscript.

Human research participants

Policy information about studies involving human research participants

Population characteristics	Blood samples were collected from the participants of both sexes, whose age ranged from 23 to 35 years. We did not test the volunteers for genotypic information and did not divide them to groups based on specific health or socio-economic features. The only inclusion criteria were the absence of severe chronic or acute diseases and the absence of medical contraindications for donating blood at the time of the study.
Recruitment	The volunteers were recruited from the employees of Pirogov Russian National Research Medical University through a public announcement. Since we used the blood samples only to collect neutrophils, which represent a model of phagocytosis, and did not compare neutrophils obtained from people with different genetic contexts or health features, we do not believe that the current study might be biased due to the procedure of recruitment. Moreover, for each experiment, all collected neutrophils were combined into a common mixture; this significantly reduces the chance that the imaged cells behaved differently in separate experiments due to some individual characteristics of the participants.
Ethics oversight	The study was approved by the local ethics committee of Pirogov Russian National Research Medical University and conducted in accordance with the Declaration of Helsinki.

Note that full information on the approval of the study protocol must also be provided in the manuscript.
